# Herpes zoster ophthalmicus with atypical occipital lobe and splenial lesions: A case report

**DOI:** 10.1016/j.radcr.2026.01.062

**Published:** 2026-02-13

**Authors:** Serena Fadell, Steve Nelson

**Affiliations:** a Wright State University Boonshoft School of Medicine, 3640 Colonel Glenn Hwy, Fairborn, OH 45324, USA; b Staff Neuroradiologist, Wright Patterson Medical Center, 4881 Sugar Maple Dr, Wright-Patterson AFB, OH 45433, USA

**Keywords:** Herpes zoster ophthalmicus, Varicella-zoster virus, Encephalitis, Splenium, Corpus callosum, Occipital lobe

## Abstract

A 32-year-old male with past medical history of migraine headaches (last episode 15 years prior) presented with persistent headache lasting 2 days and with associated nausea, photophobia and phonophobia concerning for migraine. Subsequent visits to outside facilities suggested a cortical/subcortical infarct of the left medial occipital lobe secondary to migraines. However, after continued symptoms and the subsequent development of a maculopapular rash along the right orbit and eyelid, suspicion grew that this was not a simple case of migraine induced infarct. Cerebral spinal fluid analysis showed markedly elevated VZV antibodies further raising suspicion. After a 1-week course of high-dose oral valacyclovir the rash and headache resolved. This case demonstrates an atypical pattern of CNS involvement in herpes zoster ophthalmicus, with simultaneous splenial and medial occipital lesions - an imaging pattern not commonly reported in immunocompetent adults. The findings also highlight the importance of a good clinical history and the superior sensitivity of MRI in detecting subtle varicella-zoster-related encephalitic changes when CT is nondiagnostic.

## Introduction

Herpes zoster ophthalmicus (HZO) is caused by the reactivation of latent varicella-zoster virus (VZV) specifically in the ophthalmic division (VI) of the trigeminal nerve. Its typical presentation involves a painful unilateral, vesicular rash of the face with possible eye involvement. HZO is typically a clinical diagnosis, and often imaging is not necessary based on the clinical history. In up to 2 thirds of cases, this involves a viral prodrome preceding the development of a painful maculopapular rash along the forehead and orbit [1]. The progression to VZV encephalitis is uncommon in immunocompetent patients and is estimated to be between 1 in 30–50,000 cases [2,3]. Central nervous system involvement is clinically urgent, and imaging findings play a key role in this instance. Magnetic resonance imaging (MRI) is considered superior to computed tomography (CT) in detecting pathology in varicella zoster encephalitis [4]. Early recognition supports timely antiviral therapy and appropriate consultation for the best clinical outcomes [5]. Here, we present a case of a young immunocompetent patient whose initial imaging raised concern for a cortical–subcortical infarct of the medial occipital lobe and splenium. He subsequently developed a painful periorbital rash consistent with HZO, prompting reconsideration of the diagnosis and concern for VZV encephalitis or vasculitis.

## Case report

A 32-year-old male with remote history of migraines 15 years prior (for which he takes no preventative medication) first presented to the emergency department with an acute headache, mild nausea, blurred vision, photo and phonophobia. Neurologic examination was nonfocal with normal ocular exam, no rash, and no visual deficit. A complete blood count was obtained, and results were within normal limits. No imaging was performed on this visit. He was treated with Toradol, Benadryl and Compazine with improved symptoms and was discharged with a diagnosis of recurrent migraine.

After 2 days, the patient returned with similar symptoms and similar treatment/response with referral to Neurology. Noncontrast head CT on this visit was initially interpreted as negative for acute intracranial abnormality. At the follow-up appointment with Neurology, he was started on Sumatriptan 50mg PRN for headache and remained without focal Neurologic deficit or visual defect.

**Fig. 1:**
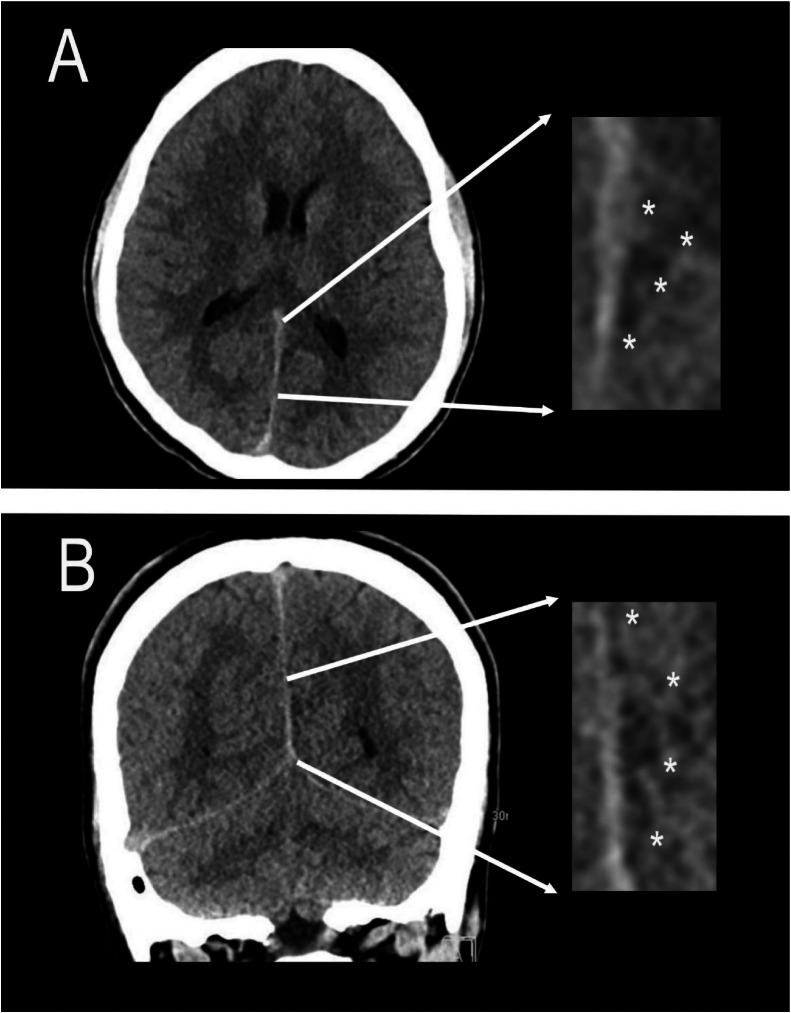
Axial noncontrast CT of the head (A) and Coronal noncontrast CT of the head (B) each with blown up field of view in the region of interest showing a very subtle triangular hypodensity in the medial left occipital lobe abutting the falx (demarcated by white asterisks) overlooked on initial imaging.

**Fig. 2:**
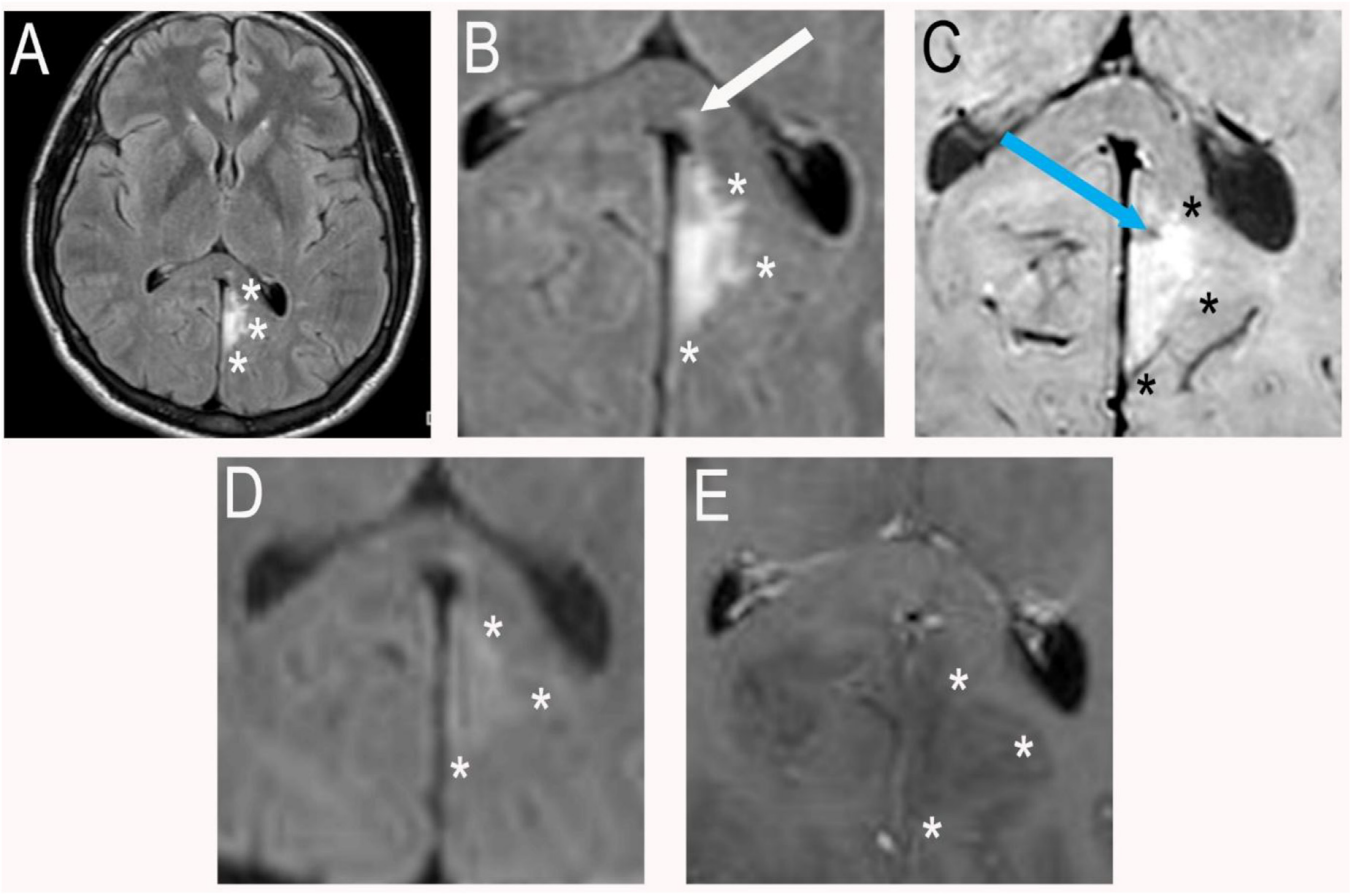
Axial T2 FLAIR (A) showing hyperintense triangular area within medial left occipital lobe and abutting the falx (white asterisks) corresponding to lesion on the prior head CT. There is also a punctate area of hyperintensity within the splenium of the corpus callosum on the cropped axial T2 FLAIR image (B) (white arrow). Cropped axial SWI (C) showing faint hyperintensity in the medial left occipital lobe (black asterisks) and punctate hypointensity anteriorly which may represent focal hemorrhage (light blue arrow). There was no diffusion restriction at the time of scanning (D) with hyperintensity corresponding to shine through (white asterisks) and Finally, axial T1 post contrast enhanced image (E) showing no enhancement of the lesion (white asterisks).

Approximately 4 weeks after the second emergency department visit, he presented to the Neurology clinic with persistent and worsening headache in addition to a mildly painful right periorbital and frontal scalp vesicular rash, clinically consistent with herpes zoster ophthalmicus. Review of his medical history showed that he had visited several local hospitals with similar complaints after the second visit to our facility and prior to the visit with Neurology. An MRI performed at 1 facility (for which data is not available) was reported to show a cortically based infarct of the left medical occipital lobe and splenium and was attributed to sequelae of migraine headache at that time. Review of noncontrast CT of the head performed at our facility on the second visit demonstrated a subtle triangular hypodensity in the medial left occipital lobe abutting the falx (Figs. 1A and B and with corresponding blown-up views of the lesion). This corresponded to MRI findings performed at our facility of a hyperintensity on T2 fluid-attenuated inversion recovery sequences (FLAIR) within the medial left occipital lobe, with an additional punctate hyperintensity in the splenium of the corpus callosum (Fig. 2A and B magnified). Susceptibility-weighted imaging showed questionable blood products anteriorly (Fig. 2C). Diffusion-weighted imaging showed faint hyperintensity without definite restriction on corresponding ADC map favoring shine through and suggesting a subacute to chronic process (Fig. 2D). Postcontrast T1-weighted imaging showed no abnormal enhancement (Fig. 2E).

Lumbar puncture was subsequently performed, and cerebrospinal fluid analysis was positive for herpes zoster antibodies over 800 (positive > 165), confirming prior infection by varicella-zoster virus. Given the presence of rash, a PCR for VZV was not performed. Additional CSF studies demonstrated a lymphocytic-predominant profile with 0% neutrophils, 96% lymphocytes, and 4% monocytes. CSF protein was elevated at 78 mg/dL with normal glucose at 60 mg/dL. Four oligoclonal bands were also present. These findings further supported an inflammatory and viral-mediated process rather than a bacterial etiology. The patient was treated with high-dose oral valacyclovir 1 g, TID for 7 days (Valtrex, GlaxoSmithKline, Research Triangle Park, NC, USA). Due to risk of subsequent stroke, he was again treated with Valacyclovir 2 g, Q6 hours for 14 additional days. During treatment his rash and well as his headache resolved.

He has had several Ophthalmology appointments given concern for long-term and possibly latent sequelae of HZO since diagnosis and has been without a visual deficit throughout the course of his illness. Follow-up MRI demonstrated a stable splenial lesion without cortical progression (data not shown).

## Discussion

Herpes zoster ophthalmicus (HZO) is caused by reactivation of VZV in the ophthalmic branch of the trigeminal nerve which can lead to ocular disease and, less commonly, central nervous system involvement including encephalitis and vasculopathy. Prompt recognition matters because antiviral therapy can reduce complications [5,6]. Diagnosis is typically made by clinical findings with a painful vesicular rash in the distribution of V1 being a chief finding. The diagnosis is supported by elevated VZV antibodies in CSF and ideally by PCR for presence of VZV. In the above case, although the CSF VZV IgG was positive, PCR would have provided higher specificity for confirming active CNS infection. Pleocytosis suggested an active viral infection, however.

This case differs from most reported HZO-associated CNS complications in that the lesions involved both the splenium and the medial occipital lobe - an atypical distribution in an immunocompetent young adult. Prior reports more commonly describe multifocal cortical infarcts or lesions at the grey-white matter junction of the cerebrum, with a majority being in the basal ganglia, cerebellum and deep white matter [7]. When in the cerebrum, the lesions are discrete, subcortical, clustered and plaque-like which typically enhance and restrict diffusion in the acute of disease [8]. Table 1 outlines the known MRI findings of those with VZV induced encephalitis and vasculitis [5,[9], [10], [11], [12], [13]]. Our findings are in keeping with the vasculopathy pattern of VZV encephalitis with a cortical/subcortical plaque-like lesion and a punctate lesion in the splenium that did not resolve over time unlike that seen with mild encephalopathy with reversible splenial lesion (MERS).

**Table 1:** MRI patterns in VZV encephalitis.

Imaging pattern	Typical location	Key MRI features	Clinical notes
Splenial lesion (reversible splenial lesion syndrome- RESLES/MERS pattern associated with VZV)	-Midline splenium of the corpus callosum- may extend to adjacent callosal body or nearby deep white matter in some encephalitis/encephalopathy cases.	-Oval or boomerang-shaped FLAIR and T2 hyperintensity in the splenium. -Marked diffusion restriction on DWI with low ADC; typically, no or minimal enhancement and no hemorrhage.	-Represents cytotoxic or intramyelinic edema related to infection, metabolic stress, or seizures in mild encephalitis/encephalopathy syndromes. -Reported with various viral encephalopathies; VZV encephalitis can rarely present with this reversible splenial pattern rather than classic vasculopathy.
Cortical/cortical-subcortical lesions (vasculopathy pattern in VZV encephalitis)	-Multiple cortical and cortical-subcortical regions at the gray-white matter junction in the cerebral hemispheres; may involve deep gray nuclei, brainstem, or cerebellum.	- Patchy or confluent FLAIR/T2 hyperintense infarcts; lesions often ovoid and well-defined. - Diffusion restriction in acute ischemic foci; variable gadolinium enhancement in subacute lesions reflecting blood-brain barrier disruption.	- Reflects viral vasculopathy with small- and/or large-vessel involvement causing ischemic stroke; hemorrhagic components and microbleeds can occur but are less common. - Seen in both immunocompetent and immunocompromised adults with VZV reactivation, often after zoster rash but sometimes without cutaneous lesions.

Our patient’s MRI showed FLAIR hyperintensity in the splenium of the corpus callosum and left anterior medial cortex, without enhancement or diffusion restriction suggesting that this process was in the subacute to chronic phase at the time of our image acquisition, 4 weeks after the initial presentation. The punctate focus of susceptibility on SWI likely reflects trace hemorrhage. Had this exam been performed at an earlier time frame, such as when the original CT was performed 1 would have expected cortical enhancement and diffusion restriction of both the occipital lobe lesion and splenial lesion. The timing is a bit odd as the purported MRI findings of encephalitis occurred before the development of the rash in the timeline. The distribution of imaging findings is also somewhat atypical in that there is involvement of the splenium. This finding has been described in several cases but is rare in the immunocompetent patient [14]. On subsequent imaging follow up 1 year later in our patient, these imaging findings were stable.

Although our patient’s initial noncontrast CT was interpreted as normal, our initial MRI performed weeks later demonstrated clear inflammatory parenchymal signal abnormalities highlighting the limited role of CT in detection of subtle parenchymal change [4,15]. Unlike herpes simplex virus encephalitis, which classically demonstrates FLAIR hyperintensity in the temporal lobes and orbitofrontal cortices, VZV encephalitis shows a broader range of imaging findings [15,16]. Recognizing these atypical MRI features is important, as MRI allows earlier detection of CNS involvement and can facilitate timely antiviral treatment.

And finally, HZO is more prevalent in the healthy elderly population (over age 50) as well as those who are immunocompromised for various reasons [17]. For example, patients with HIV or Systemic Lupus Erythematosus have an increased risk of Herpes Zoster infection and reactivation [17]. Our case is atypical as the patient was a previously healthy young male under age 50 and with no risk factors to include immunocompromised state to include Diabetes, HIV, SLE, previous cancer diagnosis and Sarcoidosis among others. Therefore, a high index of suspicion, brain MRI and CSF sampling are crucial to making a timely diagnosis particularly in those presenting with migraine headaches.

## Patient consent

Written, informed consent was obtained from the patient allowing for publication of their case and related findings.
